# High resolution building facade dataset for facade elements parsing

**DOI:** 10.1016/j.dib.2025.111950

**Published:** 2025-08-06

**Authors:** Shuchang Xu, Fan Wu, Junjie Cheng, Wenzhen Yang

**Affiliations:** a School of Information Science and Technology, Hangzhou Normal University, Hangzhou, Zhejiang 311121, China; b Zhejiang Lab, Intelligent Equipment Research Center, Hangzhou, Zhejiang, China; c College of Computer Science and Technology, Zhejiang University, Hangzhou, Zhejiang, China

**Keywords:** Facade parsing, Computer vision, Architecture

## Abstract

This paper introduces a high-resolution building facade dataset—hznu_facade, designed to provide rich data support for urban modeling, digital twin city construction, and facade analysis tasks. The dataset comprises facade images of 624 buildings from Hangzhou, China, covering diverse architectural styles from both commercial and residential areas. Additionally, it offers twin versions of the facade images with perspective correction. To ensure data diversity, the dataset includes images captured under various lighting conditions, including challenging environments such as direct sunlight and backlighting. Furthermore, the dataset places particular emphasis on the annotation of window elements, offering high-density window information that significantly improves the capabilities for architectural element recognition and analysis. During the annotation process, this paper leverages existing segmentation models to assist in the labelling of architectural elements, covering six primary categories: buildings, cars, trees, windows, skies, and doors. The dataset annotates a total of 43,277 windows, far exceeding existing related datasets, highlighting its strong potential for building analysis, architectural element recognition, and virtual reality (VR) applications. This dataset provides an valuable benchmark for facade parsing algorithms, contributing to the advancement of research and development in this field.

Specifications Table

**Table:** 

Subject	Computer Vision, Virtual Reality, Architecture, Culture Reservation, City Planning, Computer Science Applications
Specific subject area	Facade parsing, Image understanding, Semantic segmentation
Data format	Raw
Type of data	Image
Data collection	This study selected building facade images from Hangzhou, China, as the primary data source. To obtain high-resolution facade data, we employed a combination of cameras and drones to capture high-quality facade images. Additionally, we used a facade correction algorithm to perform perspective correction on images taken at oblique angles. The cameras used for image capture included the Canon M50 Mark II, while the drones - DJI Mavic Air and DJI Mavic 3- captured aerial views to provide frontal perspectives of the facades. The drones were positioned at eye level to ensure the complete outline of each building facade was captured as comprehensively as possible. However, due to the narrow spacing between some buildings, the field of view was occasionally restricted, resulting in only partial facade coverage in certain cases. In total, we collected high-resolution facade images from 624 buildings across various districts in Hangzhou. These front views are crucial for urban modelling and digital twin city construction. The camera resolutions ranged from 3 K to 4 K, ensuring sufficient detail for precise analysis. The images were captured under diverse lighting conditions, including direct sunlight, backlighting, and other lighting conditions. We also developed a specialized algorithm to generate perspective corrected façade images. This process eliminates distortions caused by oblique capture angles, resulting in two versions of the data: the original images and the perspective-corrected images. The original one contains high-resolution facade images captured from multiple angles, while the corrected version includes only the perspective-corrected images. Our dataset provides highly accurate facade representations, which can be used for detailed architectural analysis and virtual reality (VR) applications.
Data source location	Institution: Hangzhou Normal University City/Town/Region: Hangzhou, Zhejiang Province Country: China
Data accessibility	Repository name: Hznu Facade Datase Data identification number: 10.17632/k387xkyc5f.1 Direct URL to data: https://data.mendeley.com/datasets/k387xkyc5f/1
Related research article	None

## 1 Value of the Data

The proposed dataset is valuable for research in city planning, digital cities, virtual reality (VR), historic building preservation & restoration and cultural heritage digitization.

• High-resolution facade images are essential for urban modeling and digital twin city construction [1,2]. Existing façade datasets typically have lower resolutions and primarily focus on European architectural styles [3,4]. In contrast, hznu_facade provides high-resolution facade images of Asian-style buildings from Hangzhou, China, including drone-captured front views. These images offer more diverse facade analysis and texture mapping, significantly improving precise urban modeling [5,6] and VR-based building rendering.
• This dataset includes facade images with higher window density, which helps improve the detection and analysis of architectural elements such as windows. Unlike previous facade datasets, it covers a wide range of building types-from commercial districts to residential areas, including structures with or without storefronts, as well as those featuring large glass curtain walls. The dataset also provides rich, high-density semantic annotations. Furthermore, the dataset includes facade images captured under challenging lighting conditions (e.g., backlighting, sidelighting), enhancing the robustness of facade parsing algorithms in complex real-world environments.
• The dataset introduces a twin version with perspective corrected facades. To our knowledge, it is the first facade image dataset featuring perspective correction. Researchers can leverage this dataset to evaluate and compare the performance of facade parsing algorithms, including semantic segmentation and architectural element recognition. This dataset can then serve as a new benchmark for facade parsing algorithms.

## 2 Background

In application fields such as urban planning and digital twin city development, there is an increasing demand for higher accuracy and greater diversity in building facade modeling. However, existing facade image datasets face three significant limitations: First, existing dataset typically offer maximum resolutions no >2K×2 K, failing to meet high-resolution requirements. Second, all samples are captured using digital cameras. Due to the height of buildings, the captured images often suffer from perspective distortion. Third, existing datasets lack samples of fully glass curtain walls, which are common in Asian urban architecture. To address these gaps, we aim to construct a new high-resolution facade image dataset, including perspective corrected versions of the images. Moreover, for the first time, we include drone-captured data in the facade dataset.

## 3 Data Description

The dataset consists of two versions: hznu_facade_original and hznu_facade_perspective_correction. The hznu_facade_original subset contains 624 records, totaling 1872 files. Each record includes original JPG images, annotated JSON files, and TXT files containing the homography matrix H used for perspective correction. The homography matrix H is typically defined as in Eq. (1). The elements of the 3 × 3 matrix are stored in the TXT file column-wise. Each column corresponds to a specific set of transformation parameters. The first column (*h*_11_, *h*_21_, *h*_31_) controls the horizontal (x) coordinate transformation. The second column (*h*_12_, *h*_22_, *h*_32_) controls the vertical (y) coordinate transformation. The third column (*h*_12_, *h*_23_, *h*_33_) encodes translation and normalization parameters of the image.

(1) $$\left[\begin{array}{ccc} h_{11} & h_{12} & h_{13}\\ h_{21} & h_{22} & h_{23}\\ h_{31} & h_{32} & h_{33} \end{array}\right]$$

**Fig. 1:**
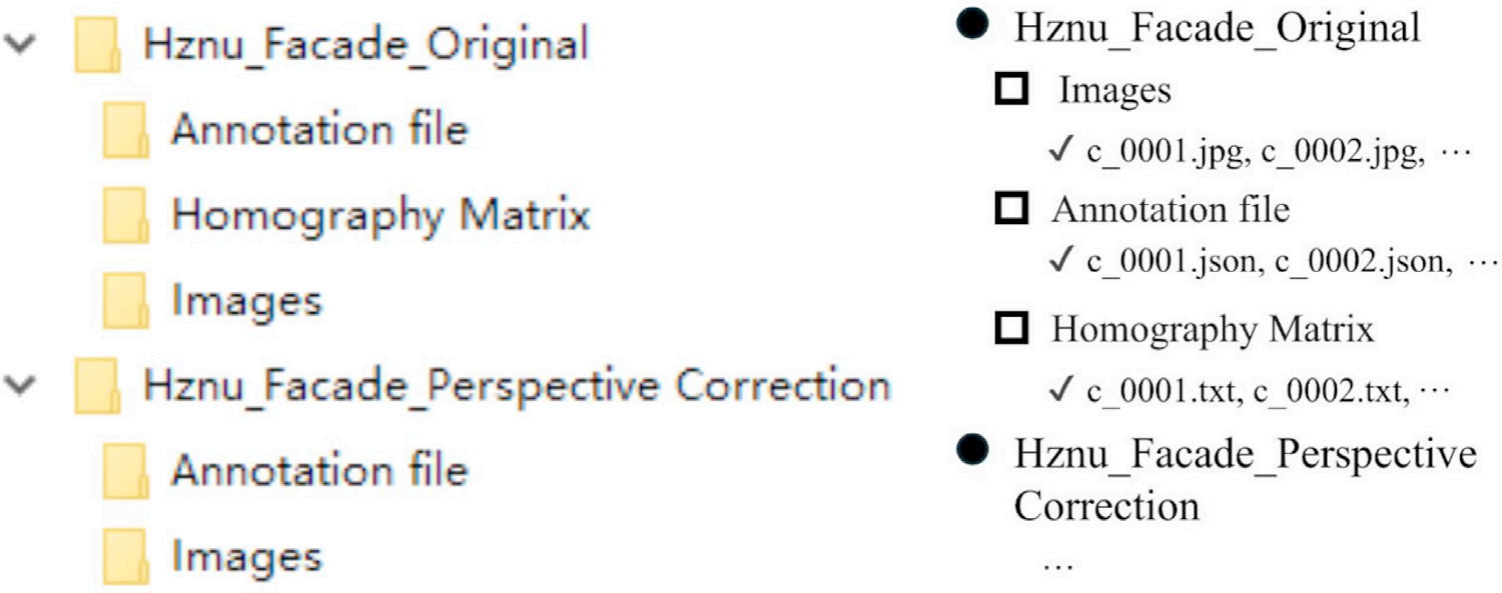
The folder structure of hznu facade origin. (left) Folder tree structure of our dataset. (right)File details in each folder.

The hznu_facade_perspective_correction version also contains 624 records, with each record consisting of perspective-corrected JPG images and their corresponding annotated JSON files. The data organization in hznu_facade_original is illustrated in Fig. 1, while hznu_facade_perspective_correction follows an analogous structure.

**Fig. 2:**
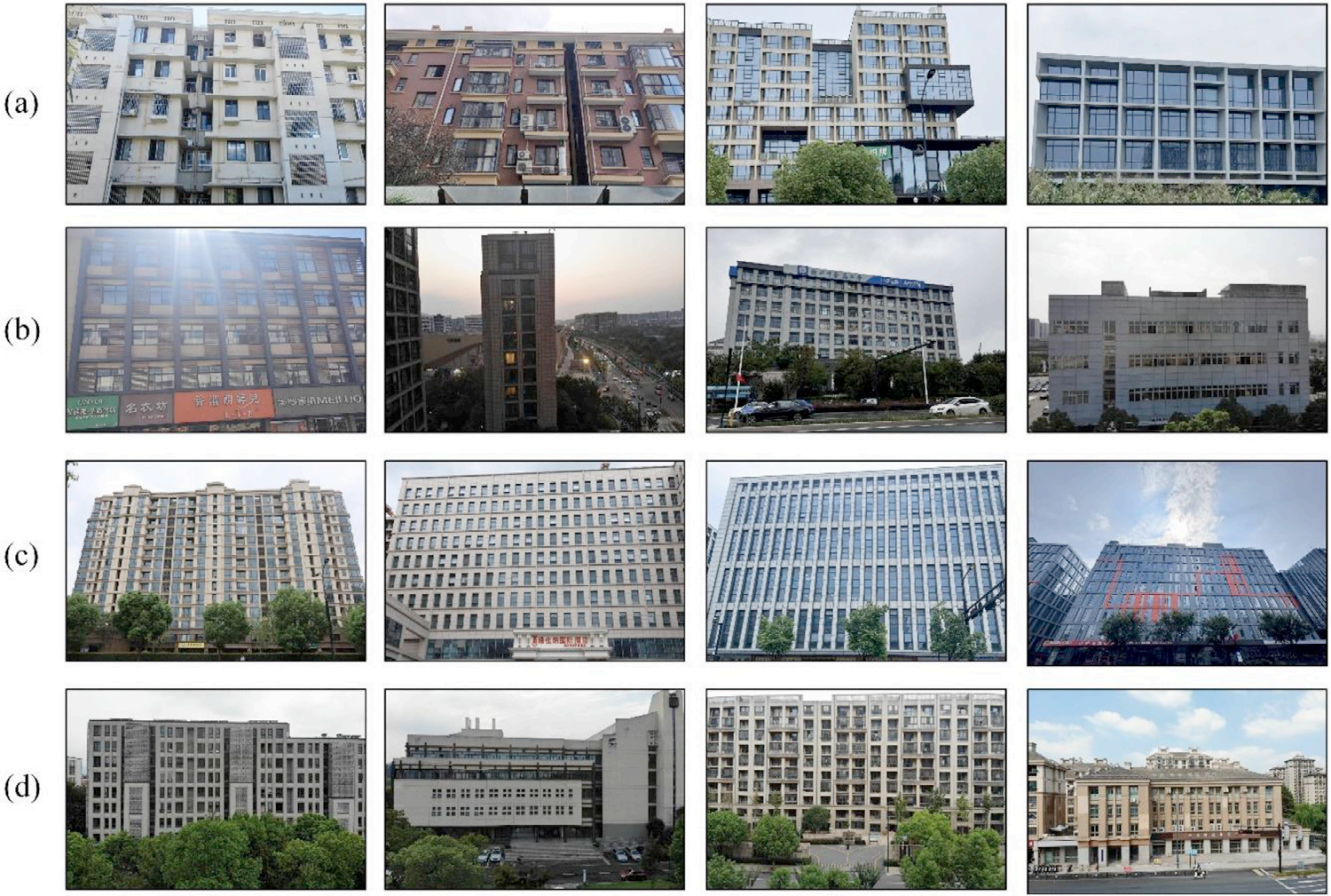
Typical samples of different facade types in our dataset: (a) facades in residential and commercial districts, (b) facades under varying lighting conditions, (c) facades with dense semantic information, and (d) facades captured by drones.

The dataset comprises facade images of various building types, encompassing a diverse range of architectural styles from residential to commercial areas. In Fig. 2: (a) presents typical facade examples of residential and commercial buildings in the Asian architectural style, illustrating representative building appearances in these regions; (b) shows facade images captured under different lighting conditions, covering a spectrum from strong to weak ambient light; (c) displays samples with high-density semantic information, highlighting rich details and structural characteristics. This diversity of sample types significantly enhances the expressiveness of the dataset and broadens its potential applicability across various scenarios.

**Fig. 3:**
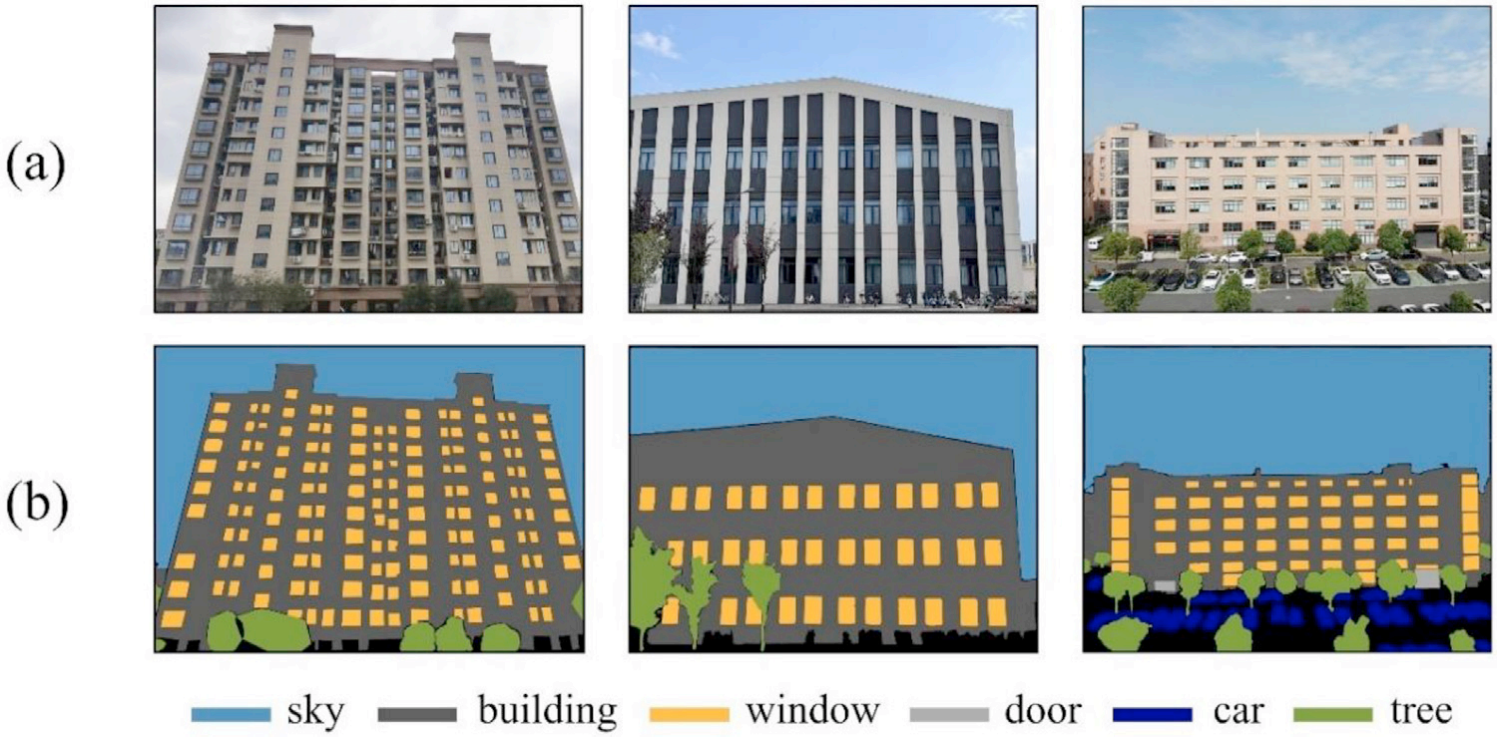
The image shows the original facade image and the corresponding segmentation map generated based on JSON file. (a) the facade image, (b) the segmentation map.

The annotation information of facade images is recorded through multiple JSON files, each containing the category and its corresponding polygonal bounding box coordinates. The corresponding segmentation maps can be generated based on the JSON files, as shown in Fig. 3.

## 4 Experimental Design, Materials and Methods

**Fig. 4:**
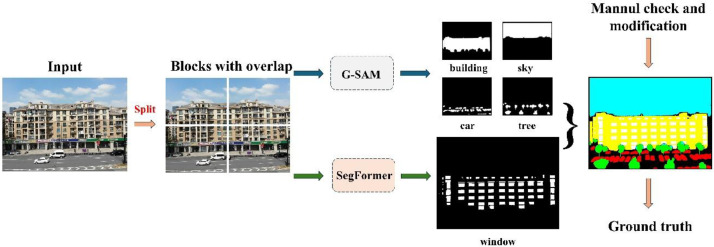
Workflow for facilitating facade image annotation. The input image is first divided into four overlapping blocks. Each block is processed separately through G-SAM and SegFormer models to obtain segmentation maps of different elements. These maps are then merged to generate the final segmentation map. After manual review and necessary modifications, the final ground truth is generated.

We annotated six categories in the facade image: buildings, cars, trees, windows, skies, and doors. Since annotating facade images is typically a labor-intensive and time-consuming process, we developed an automated workflow to improve efficiency, as shown in Fig. 4.

To fully leverage the high-resolution capabilities of our facade images, we divided each image into four overlapping regions to provide more precise annotation references. Our experiments indicate that different models excel at extracting different elements: SegFormer [12] performs exceptionally well in window detection, while G-SAM [13] is superior in segmenting other objects such as buildings, cars, and trees. To maximize the strengths of these models, independent models are applied to each region to extract different facade elements. The results are then combined to generate the initial segmentation map, which is further refined and optimized through manual checks to produce high-quality final annotations.

Statistical analysis of the dataset annotations reveals that the dataset contains particularly rich information on windows. As shown in Table 1, the dataset records a total of 43,277 windows, with an average of 69 windows per image, significantly surpassing existing datasets. This abundance of window information makes our dataset highly valuable and applicable for facade analysis, architectural element recognition, and research related to urban planning.

**Table 1:** Comparisons of our dataset with existing datasets in terms of images count, average windows per image and data type.

Dataset	Number of Images	Image Resolution	Total Window Count	Average Windows per Image	Data Type
ECP 2010 [7]	*104*	*1024×768*	*2976*	*28*	*Camera photography, European street buildings*
RueMonge 2012 [8]	*428*	*800×1067*	*8416*	*19*	*Camera photography, European street buildings*
ENCP 2016 [9]	*79*	*1280×960*			*Camera photography, European street buildings*
CFP 2024 [10]	*602*	*2560×1440*	*12,048*	*20*	*Camera photography, European and Asian architecture*
Hznu Facade 2025 [11]	*624*	*4059×3956*	*43,277*	*69*	*Camera photography, drone photography, Asian architecture*

**Fig. 5:**
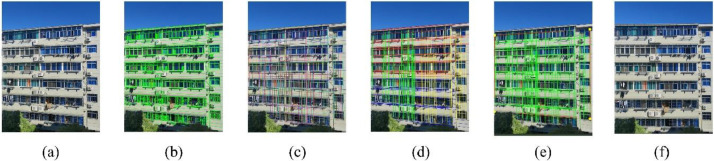
Perspective correction flowchart. (a) Original image; (b) Straight line detection; (c) Line classification and fitting; (d) Region division; (e) Intersection point calculation; (f) Image after perspective correction.

To generate perspective-corrected facade images, we developed an automated algorithm for homography matrix computation, which comprises five key steps, as illustrated in Fig. 5*:*

(1) Straight Line Detection: Image processing algorithm is applied to detect all straight line segments in the facade image.
(2) Line Classification and Fitting: Detected line segments are clustered based on their slopes and intercepts. Segments with similar parameters are grouped (indicated by uniform colors in Fig. 5c) and fitted into longer, structurally complete lines.
(3) Region Division: The image is partitioned into four regions—upper, lower, left, and right—with distinct colors in Fig. 5d denoting the line segments within each region.
(4) Intersection Point Calculation: Boundary lines are extracted from each region, and their intersections are computed. The four resulting points define the vertices of the largest quadrilateral enclosing the façade area.
(5) Perspective Correction: A homography matrix can be easily derived from the quadrilateral’s vertex coordinates. Applying this matrix yields the perspective-corrected image.

*Here is an example code:*

Import numpy as np
image = cv2.imread ('input.jpg')
*H* = np.loadtxt ('homography_matrix.txt')
height, width = image.shape[:2]
corrected_image = cv2.warpPerspective (image, H, (width, height))

**Fig. 6:**
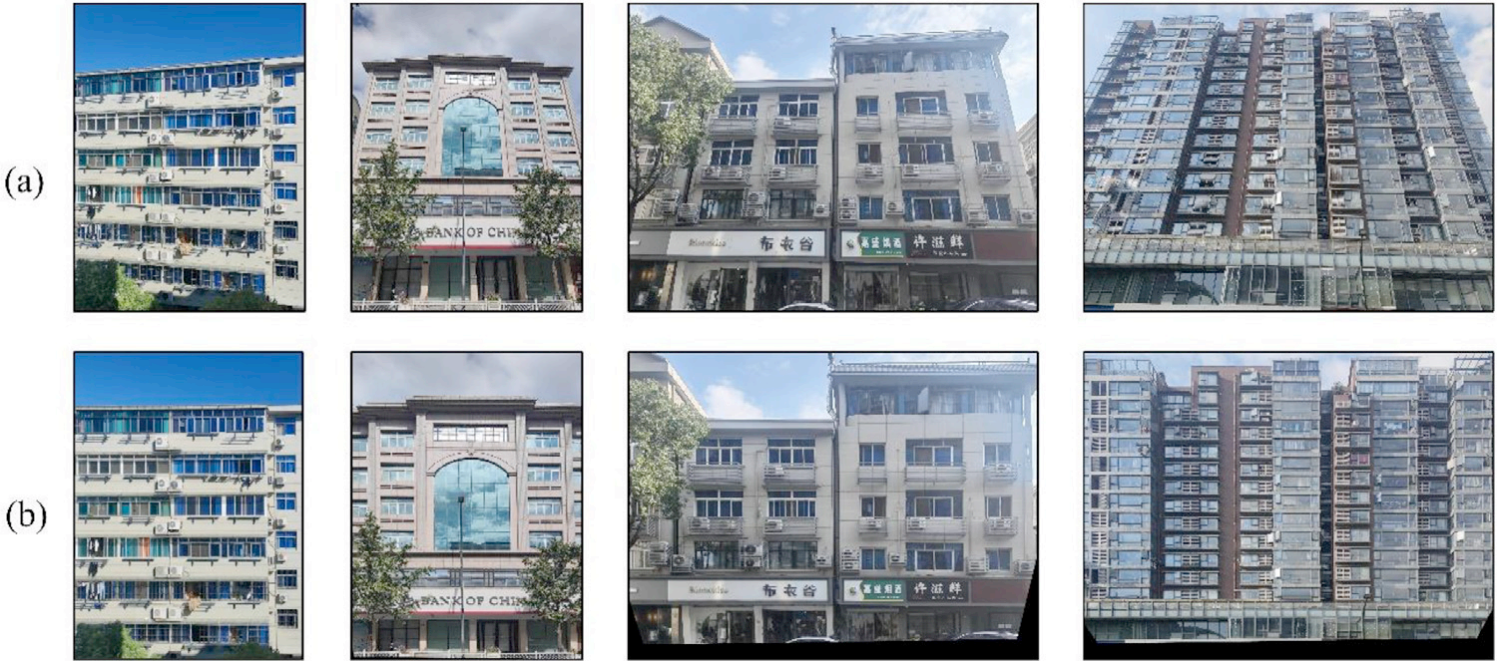
Comparison of images before and after perspective correction: (a) original facade image captured from a side view; (b) corresponding facade image after perspective correction.

Additionally, we offer a twin version of the facade image dataset featuring perspective correction, which is particularly valuable for applications such as texture mapping and culture heritage reservation. To the best of our knowledge, this is the first publicly available facade dataset with perspective-corrected samples. Each original image is paired with its corrected counterpart, as shown in Fig. 6. These comparative samples clearly demonstrate the transformation of facade angles through perspective correction, enabling more accurate utilization in 3D modelling and related applications.

**Fig. 7:**
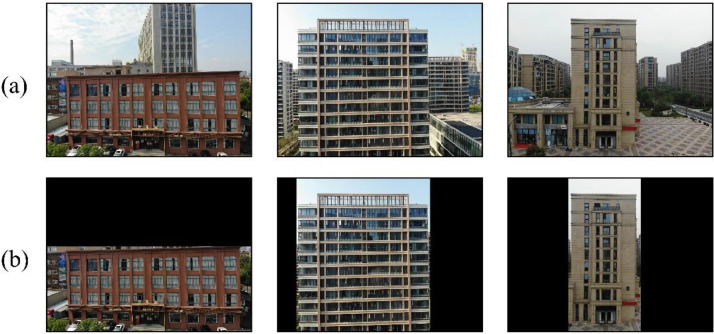
Comparison of images before and after multiple buildings process: (a) original facade image; (b) corresponding facade image after background labelling.

While most images in our dataset focus on a single dominant building, a subset contains multiple structures within a single image. To ensure clarity, we manually masked non-dominant regions (such as background elements) by labelling them as black, as shown in Fig. 7.

## Limitations

It took us several months to navigate various city blocks to capture facade images with diverse styles. Data acquisition is extremely time-intensive due to the complexities of drone-assisted photography and the laborious nature of manual image annotation. As a result, the number of samples in our dataset is comparable to existing datasets.

Regarding annotation quality, minor unlabelled areas may occasionally persist at class boundaries—a limitation shared by most facade datasets. Additionally, we observed geometric distortions in some drone-captured images (specifically from the DJI Mavic 3). Deformed window shapes can be found in samples d0241, d0239, d0224, d0221, d0216, d0113, d0107, and d081.

## Ethics Statement

The authors have read and follow the ethical requirements for publication in Data in Brief. We confirmed that no human subjects were involved in this study. Therefore, informed consent and ethical committee approval were not required. Animal experiments were not conducted. Data collected from social media platforms was not used in this research. Therefore, participant consent and data redistribution policies were not applicable.

## Declaration of Competing Interest

The authors declare that they have no known competing financial interests or personal relationships that could have appeared to influence the work reported in this paper.

## Data Availability

Mendeley DataHznu Facade Dataset (Original data).
